# Perspectives on and Experiences With Bullying From Youth With Neuromuscular Conditions

**DOI:** 10.1177/08830738241257985

**Published:** 2024-06-11

**Authors:** Nurin Chatur, Christina Ippolito, Laura McAdam

**Affiliations:** 1 37205 Holland Bloorview Kids Rehabilitation Hospital, Toronto, Ontario, Canada; 2 Bloorview Research Institute, Holland Bloorview Kids Hospital, Toronto, Ontario, Canada; 3 Department of Pediatrics, 12366 Temerty Faculty of Medicine, University of Toronto, Toronto, Ontario, Canada

**Keywords:** Duchenne muscular dystrophy, myopathy, pediatric, quality of life

## Abstract

**Aim:** To understand the bullying experiences of youth with neuromuscular conditions. **Method:** Fourteen participants with neuromuscular conditions (10 male; 10-19 years old) participated in semistructured interviews that were analyzed using inductive thematic analysis. **Results:** Four overarching themes were identified: (1) participants experienced stigma-based bullying; (2) participants exhibited resilience despite bullying victimization; (3) participants identified personally and theoretically helpful and unhelpful supports with regard to bullying; and (4) participants proposed bullying interventions. **Interpretation:** Individuals with neuromuscular conditions had unique experiences and perspectives on bullying. This qualitative study provides health care professionals with insight into the bullying experiences of patients with neuromuscular conditions. Findings highlight the role for formal and informal education to mitigate stigma-based bullying and increased opportunities for peer support as a protective factor against bullying.

Children and youth with physical disabilities are more likely to experience bullying than those without a disability.^1,2^ Children with chronic physical illness or disability were found to be more likely to be victims of all types of bullying, including physical (eg, pinching, hitting; odds ratio [OR] = 1.47), social (eg, spreading rumors, exclusion; OR = 1.47), verbal (eg, teasing, name calling; OR = 1.67), cyberbullying (eg, using social media or text messages to bully; OR = 1.29), and illness-specific teasing (OR = 5.29), specifically if the illness was visible.^1^ Children with neuromuscular conditions, which are a group of disorders causing muscle weakness that are often progressive, are thought to be at an elevated risk of experiencing bullying. For example, 43% of individuals with Becker muscular dystrophy reported experiencing bullying,^2^ which is greater than the reported rates in large school-based studies reported at approximately 30%.^3^

Bullying is defined as unwanted aggressive behavior that involves an actual or perceived power imbalance, is repeated or likely to be repeated, and may cause harm or distress to the person being bullied.^3^ Bullying often occurs in the context of a victim living with or perceived to be living with certain identities, characteristics, or attributes that are perceived to be socially devalued.^4^ This type of bullying is known as stigma-based bullying, which is the overlap of bullying and discrimination whereby discrimination is defined as a behavioral manifestation of social devaluation.^4^ It has been suggested that victims of stigma-based bullying have worse mental health outcomes than victims of non–stigma-based bullying.^5^

Bullying as a whole has significant short-term and long-term impacts on victims, including decreased physical and psychological well-being, reduced academic functioning, and increased risk of mental health conditions, and it has negative impacts on peer and parental relationships.^6–9^ In a systematic review of school-aged children and adolescents, all studies reported that victims of bullying experienced a decline in health-related quality of life.^7^ The effects of childhood bullying victimization are far reaching and have been linked with poorer health outcomes as an adult, including higher rates of anxiety, depression, and suicidality.^10–12^

Resiliency, defined as the attainment of positive outcomes, adaptation, or developmental milestones in spite of significant adversity, has been linked with improved bullying outcomes.^13^ Resiliency can be further disaggregated into a factorial model with 3 personal protective factors: (1) mastery (optimism, self-efficacy, adaptability); (2) relatedness (trusting others, feeling comforted by others, feeling that one's differences will be accepted); and (3) emotional reactivity (sensitivity to an adverse event, recovery from the event, impairment that the event creates in an individual).^14^

Currently, understanding of the bullying experiences of youth with neuromuscular conditions and the factors that increase or decrease the impact of bullying on these youth is limited. Therefore, the objective of this study was to explore the bullying perspectives on and experiences of youth with neuromuscular conditions.

## Patients and Methods

This study used an exploratory, descriptive qualitative design as part of a larger cross-sectional, multicenter, mixed methods study. A survey was administered at a single time point to participants and optionally to parents. Participants were invited to complete a semistructured interview, which was the focus of this study. All participants, including caregivers (if the participant consented to their involvement), provided written informed consent. The study was approved by the Research Ethics Board at Holland Bloorview Kids Rehabilitation Hospital in Toronto, Canada (REB 0201) and the Children's Hospital of Eastern Ontario in Ottawa, Canada (REB 20/132X).

### Participants

Participants were recruited from 2 pediatric neuromuscular clinics in Ontario, Canada. Inclusion criteria included (1) 10-19 years of age, (2) have a neuromuscular condition, (3) able to read and communicate in English, and (4) consent to participate in the semistructured interview. There were no exclusion criteria associated with this study.

### Procedure

Twenty-three participants were invited to complete an interview and 14 participated in semistructured interviews over secure video conference between November 2021 and October 2022. Interviews were conducted by N.C., a female-identifying neurologist with experience working with young people with neuromuscular conditions. Participants had no prior relationship with the interviewer.

Interviews opened with a definition of bullying; then questions covered demographic information about the participant and general questions about the participant's experiences with bullying. More specific questions followed designed to address gaps in understanding youth with neuromuscular conditions’ perspectives on bullying, including why they think children and youth are bullied, who they felt was responsible to help deal with bullying, and coping strategies and supports they found helpful. Interviews were audio-recorded and transcribed verbatim for analysis.

### Data Analysis

Inductive thematic analysis was conducted, following the steps outlined by Khan and Glaser and Straus for a grounded theory approach.^15,16^ Two team members (N.C., C.I.) reviewed the transcripts to familiarize themselves with the data and then created a living codebook. Interviews were coded independently and then compared. Discrepancies were resolved through team discussion with all team members (N.C., C.I., L.M.). Codes were grouped into broader subthemes, which then defined overarching themes. When additional themes were identified during the data analysis and collection process, earlier transcripts were reanalyzed. A final consensus was reached between all team members. Field notes and debriefs of discussion were kept to enhance trustworthiness of the analysis and ensure dependability of the findings. Recruitment, interviewing, and analysis occurred concurrently until data saturation was reached and no new concepts were discussed within interviews.^17^ Data analysis was completed using NVivo (version 12.0).

## Results

Fourteen youth participants with neuromuscular conditions completed interviews that were 20-60 minutes in length. Eleven caregivers joined the youth in interviews. Youth participants’ mean age was 14 years (SD = 3 years 2 months; R = 10-19 years). Ten youth participants identified as male and 4 identified as female. Seven youth participants used a mobility device. All youth participants (n = 14) attended school in a typical classroom and 3 youth participants also indicated they were part of a special education classroom. Youth reported having between 1 and 20 friends. See Table 1 for additional demographic information.

**Table 1.** Youth Participant Demographics.

Clinical characteristics	n (%)
Diagnosis	
Duchenne muscular dystrophy	7 (50)
Myotonic dystrophy	3 (21)
Congenital myopathy	1 (7)
Congenital muscular dystrophy	1 (7)
Spinal muscular atrophy	1 (7)
Congenital myasthenic syndrome	1 (7)
Comorbidities	
Learning disability	5 (36)
ADHD	2 (14)
Intellectual disability	1 (7)
Anxiety/mood disorder	2 (14)
Ethnic Group	
South Asian	3 (21)
Filipino	2 (14)
White	8 (58)
Southeast Asian	1 (7)

Abbreviation: ADHD, attention-deficit hyperactivity disorder.

Four overarching themes were identified from the interviews: (1) participants experienced stigma-based bullying; (2) participants exhibited resilience despite bullying victimization; (3) participants identified personal and theoretical helpful and unhelpful supports with regards to bullying; and (4) participants proposed bullying interventions.

### Theme 1: Stigma-Based Bullying

Participants reported being discriminated against because of their physical differences related to their underlying neuromuscular condition. Additionally, participants identified other differences as the source of their bullying victimization that they perceived to be socially devalued, such as differences in weight or stature, speaking English as a second language, and differences in their voice. These traits were noted to impact their experiences at an individual perpetrator level, an interpersonal level, and a structural level involving their environment and institutional policies. Four subthemes were identified.

#### Social Stigma

Participants described that their differences perceived as socially devalued made them more likely to be victims of bullying. Examples of these differences included requiring a mobility support, not being able to speak loudly because of requiring a tracheostomy, having English as a second language, and having differences in their body weight.

With my meds, it halted my growth for a really long time. I didn’t gain weight and yeah, I was definitely teased about that for a while, and even my facial expression. (P11, youth)

#### Individual Perpetrator

Participants endorsed that some of the individual perpetrators engaged in a social dominance orientation because of the participants’ perceived socially devalued characteristics.

Because [children with neuromuscular conditions] are usually different than normal kids and people are afraid of that, so they make them feel low so they can feel high. (P3, youth)

#### Interpersonal

Participants described that often these negative interpersonal interactions were driven by the perpetrator not understanding the victim's differences and how to include them in peer activities. For example, participants were not invited to friends’ events (eg, ice skating, swimming) or to play during school breaks because others did not believe they could participate in the activities. Participants did not believe their friends intended to hurt them, but their feelings were hurt, nonetheless.

If I can walk, why am I in a wheelchair? So they were just laughing at that. . . . All the time if I am in a new class. (P2, youth)

I don’t do many things after school with my friends, except in the summer. And I found that people wouldn’t invite me over because they would be saying well, I mean, we can’t like, like my parent said like, “Hmm. I’m going to go swimming so I mean, I don’t think we can invite her.” Yeah, they were aware of it, but I don’t think they purposely intended to hurt me. I just think they weren’t thinking about my feelings in a way. (P10, youth)

#### Structural

Participants reported structural stigma manifestations leading to stigma-based bullying, including in the school environment and school policies. For example, schools that did not have equitable accessible entrances created potential and actual bullying situations for participants from peers and teachers.

Even an [occupational therapist] recommended school to have a sitting or bench, especially outside, you know, in the area where such kids can sit if they are tired. But unfortunately, you know, the school did not bother to pay attention. (P7, caregiver)

### Theme 2: Resiliency

Participants endorsed that over time through repeated bullying events, peer support, and/or through external resilience building programs, many of the participants were able to persevere when encountering bullying.

#### Mastery

Participants felt capable in their ability to problem solve when they were bullied. Particularly, through victimization experiences, participants adapted new strategies to manage bullying incidents. For example, instead of focusing on negative emotional reactions, participants sought solace in their peers and family, ignored the bullying, or stood up for themselves. Participants had positive attitudes about the world and their life despite experiencing bullying, and participants were flexible in their approaches in dealing with bullying over time.

I basically just talked about it with [my parents], and I tried to like, find solutions on my own, because I don’t like asking people to help me do things. So, if I find a solution myself, good for me. (P10, youth)

#### Relatedness

Participants perceived that having trusted supports around them, including their family members, peers, and teachers, who were reliable and accepting of them would support them when facing bullying. Participants indicated that sometimes they would reach out to these trusted individuals for support, and other times the supporter initiated the contact with them. Participants endorsed feeling comforted by those around them and that these formal and informal support groups understood their differences. Furthermore, participants found it helpful to understand why bullies may bully to increase their tolerance to potential negative interactions. For example, some participants rationalized that perpetrators may be facing their own challenges, and that the perpetrator may be taking out their frustration on the participant, rather than bullying incidents being the participants’ fault.

I feel like I responded positively [to my mom's explanation of bullying] because I just thought, like, I start understanding why some people might bully. And like, you know, if I’m younger I might not know these things, and so, just like excellent explanations really helped to like cope, and like understand why these things are happening. (P1, youth)

#### Emotional Reactivity

Some participants described having decreased sensitivity to bullying incidents over time, whereas others reported ongoing sensitivity and impairment when encountering bullying. Over time, some participants reduced their reactivity to bullying by deciding they did not care, laughing off the experience, distracting themselves, and attending coaching sessions to build resilience. However, some participants reported ongoing reactivity to bullying situations where they endorsed feeling upset. Other participants also reported being unable to regulate their emotions and reacting physically in some situations.

### Theme 3: Unhelpful and Helpful Supports

Participants reported that helpful support included their school, peer support, parents, and external support through health care institutions. It is important to note that most of the helpful supports described by participants were driven by the participant and their family. Participants also reported unhelpful supports that were primarily related to institutions.

#### Helpful Supports

A commonly described helpful support was educating the participants’ peers about their condition. Participants did a presentation at the start of the school year or when significant changes occurred to educate those surrounding the participant about the participant's neuromuscular condition, including what it was, what makes them different, and with what the participant may need help. One participant and their caregiver described having the participant's peers being able to ask questions about the participant's condition as positive. Helpful supports to minimize bullying also included having strong peer relationships, parental support, and having teachers and principals that provided emotional support and acted proactively or swiftly in response to bullying.

I talked to my mom and my friends are really supportive [which has helped me with bullying]. (P3, youth)

#### Unhelpful Supports

Participants endorsed that at times, supports thought to be helpful, like peer support or teachers, could sometimes not be helpful. For example, participants described that teachers were often unable to witness bullying incidents and thus were not able to intervene during the bullying event or after for preventative or punitive action.

You know, it's very often nobody around us [during school], so it's nobody can notice [the bullying]. (P1, youth)

### Theme 4: Proposed Future Interventions and Helpful Strategies

Participants discussed actions that worked for them when bullied or what they think could be done better for their community. Participants and their caregivers proposed future interventions for youth and their families to reduce bullying incidents as well as the effects of bullying, including building resiliency, developing peer social supports, enabling self-advocacy, and educating peers and teachers. Table 2 provides a list of potential strategies for youth, families, school staff, and clinicians.

**Table 2.** Intervention and Prevention Strategies Proposed by Youth Participants for Target Groups.

Target group	Recommendations if bullied	Recommendations for bullying prevention
Youth with NM conditions	Tell someone you trust like a teacher, parent, or friend Remove yourself from the situation and calm yourself down Ignore the bully If excluded, suggest an activity in which you can participate	Encourage people around you to ask questions about your condition to educate them Use a buddy system Participate in coaching or goal-based programs to help deal with bullying Join clubs to help make friends
Families of youth with NM conditions	Be an open listener and talk to your child about bullying Talk to teachers early so smaller incidents do not become targeted, persistent bullying Stay positive and empower your child to deal with bullying, such as stay calm, tell a teacher, and reminding your child of their worth	Present about your child's condition to their class at the start of the school year to help their peers build empathy Talk to teachers about concerns you have about bullying and exclusion Ensure parents of your child's friends understand the accommodations they need so they are not left out of group activities Involve your child in group activities to help them build friendships
Clinicians	Use solution-based coaching to build goals with clients and families about coping with bullying Discuss bullying with patients and families early Provide families with recommendations from this report	Suggest presenting about one's disability to their class Promote families in helping children develop peer support
Schools	Stand up for students being bullied Promote open dialogue between students so that they do not leave someone out because of accessibility concerns Set up an interview with families and children involved in bullying in a safe space Be open to hearing students’ experiences and do not make assumptions	Set up an anonymous tip line for students too shy to ask for help directly from an adult Set up buddy systems so everyone has someone looking out for them Talk to all students about bullying and focus on building empathy Normalize differences

Abbreviation: NM, neuromuscular.

## Discussion

The aim of this study was to better understand the bullying experiences faced by youth with neuromuscular conditions. Youth with neuromuscular conditions are the experts of their lived experiences and, therefore, were best able to provide insight into their experiences and protective and unprotective factors when they experience bullying, and the most helpful supports. Thus, the findings of this study fill gaps in both the neuromuscular and bullying fields, and provide relevant information across neuromuscular diagnoses, mobility statuses, and ages. The participants of this study encountered unique bullying experiences.

A common theme throughout the participants’ experiences was the phenomena of stigma-based bullying. Many participants explained that they were bullied because of a characteristic they identified as being socially devalued and was associated with their physical disability. Interestingly, participants noted that educating their peers about their neuromuscular condition often prevented, reduced, or stopped bullying victimization. Participants felt that these educational opportunities, particularly when in a group setting (eg, class presentation) prior to any bullying incidents, created an environment where their peers were able to ask questions about their disability to clarify misconceptions about disability and empathize with the participant. Providing educational opportunities to supportive peer groups allowed participants’ peers to learn about the participant's needs and to also empower these same peers to take initiative to support the participant at school and in the event of bullying incidents. Previous studies reported that formal peer counseling and support was an effective bullying intervention.^13^ Additionally, peer counseling may be a cost-effective method in schools to minimize bullying impacts and improve school culture.^18^ For children and youth with disabilities, the study findings are in line with current literature, which states that education about socially devalued characteristics such as disability and bullying interventions that directly address stigma help address bullying around disability.^4^

Participants endorsed features of the factorial model of resilience (ie, mastery, relatedness, and emotional reactivity) established by Moore et al.^13^ Participants reported that these resiliency factors helped support them when they experienced bullying, particularly endorsing the feeling of mastery. Previous studies state that students with higher resilience were less distressed by bullying victimization.^14^ In the current study, many participants described that they built up resilience over time despite, or perhaps because of, bullying victimization. However, to avoid putting the onus on bullying victims to gain mastery through their experiences of bullying, increasing opportunities for relatedness (eg, social skills programs, peer support programs, and teacher mentor programs) in schools can be a proactive approach to build resiliency as a schoolwide bullying prevention intervention.^14^ Additionally, the findings from this study suggest that further research is needed to explore resiliency building in youth with chronic conditions as it may be a protective factor to other adverse experiences.

All youth participants in the study have neuromuscular conditions, which are chronic conditions associated with a physical disability. Bullying literature commonly combines individuals with several types of medical diagnoses, and findings are applied across groups.^1,19^ We believe the findings from this study are generalizable across neuromuscular conditions, individuals with chronic conditions, and individuals with physical disabilities. For example, stigma-based bullying (theme 1) may happen to anyone in an event where a bullying perpetrator deems the individual to have a “socially devaluing characteristic.” The literature has shown that individuals with both physical and chronic conditions tend to experience higher rates of bullying,^1,19^ both of which are associated with neuromuscular conditions. Helpful and unhelpful supports (theme 3) described by the youth participants, as well as the recommended interventions and strategies (theme 4) such as classroom presentations and building relationships for positive support networks are relevant across the childhood disability field in general. Further research into resiliency in the face of bullying across youth groups must be completed.

Furthermore, the findings of this study are important and relevant for clinical practice as many participants and their families directed the bullying-related interventions they used. Clinicians are an integral support for families with neuromuscular conditions, and their involvement are needed to minimize the burden on families since significant caregiver burden already exists.^20^ Participants reported experiencing bullying, specifically stigma-based bullying, and often faced emotional challenges in responding to these bullying experiences. This emphasizes the need for clinicians to be aware of bullying risks in the neuromuscular population. It is recommended that clinicians keep an open dialogue with patients and families about bullying, as well as the facilitators and barriers that others with similar experiences have faced. This discussion can be integrated into clinical visits as a part of screening as it is known that children and youth with chronic conditions are at higher risk of being bullied.^1,2^ It is recommended that children, youth, and families be educated about bullying, and the coping and intervention suggestions from participants in this study shared with neuromuscular clients. Furthermore, there is an overall lack of literature exploring bullying and the barriers and facilitators in many physical disability populations. The suggestions from this study may be helpful to all individuals with physical disabilities, and particularly those with progressive muscle weakness like the sample included in this study.

Although the objectives of the study were met, limitations exist. Participants were required to be proficient in English, which may have resulted in the underrepresentation of families who speak other languages. In the future, completing interviews with interpreters may increase the reach of the findings. Additionally, self-selection bias may have occurred and impacted the generalizability of these themes as the youth who completed interviews may have had greater reflections on their resilience in the face of bullying, self-efficacy in their family's approach to bullying prevention, and therefore, may have had different experiences with bullying. Regardless, participants shared their experiences from the perspective of individuals with neuromuscular conditions and provided insights and suggestions that could be applicable to all members of the community.

## Conclusions

The findings from this study resulted in a better understanding of the bullying experiences, particularly stigma-based bullying, of youth with neuromuscular conditions. Participants were bullied intentionally and unintentionally. Suggestions and recommendations were provided by youth for youth, families, schools, and clinicians. Formal and informal education about an individuals’ condition and support required for individuals with neuromuscular conditions, or physical disabilities in general, may be helpful in reducing bullying incidences. Clinicians are an integral part of the support network for youth and their family and should be a resource for patients and families when facing and addressing bullying.
